# Lemierre Syndrome Presenting as Tender Goiter

**DOI:** 10.7759/cureus.51023

**Published:** 2023-12-24

**Authors:** Nishtha Manuja, Sourya Acharya, Sunil Kumar, Shanli Parnia, Anshul Sood, Stephanie Jangha

**Affiliations:** 1 Medicine, Jawaharlal Nehru Medical College, Wardha, IND; 2 Internal Medicine, Cimpar SC, Chicago, USA; 3 Radiodiagnosis, Jawaharlal Nehru Medical College, Wardha, IND; 4 Internal Medicine, Ross University School of Medicine, Florida, USA

**Keywords:** ijv thrombosis, lemierre syndrome, fusobacterium necrophorum, thyroid swelling, goiter

## Abstract

Internal jugular vein (IJV) thrombosis, also known as Lemierre syndrome (LS), is a potentially dangerous complication that follows oropharyngeal infections. It has also been documented in individuals with cervical lymph node infection, thyroid abscess, and pharyngeal abscess. LS is potentially a catastrophic complication and, if not detected and treated early, can lead to mortality. This case report describes an older woman who presented with a history of fever, odynophagia, and swelling in the anterior aspect of her neck for 20 days. Examination revealed a severely congested posterior pharyngeal wall with bilateral tonsillitis and a tender goiter. Further investigations revealed a diagnosis of LS. The patient was appropriately managed with higher antibiotics and anticoagulation. This case report highlights the importance of IJV thrombosis, which can present as a thyroid swelling.

## Introduction

Dr. André-Alfred Lemierre initially identified Lemierre syndrome (LS) in 1936 as internal jugular vein (IJV) thrombosis, which is more typically caused by a complication of an oropharyngeal infection and less commonly caused by mastoiditis or a tooth infection [1]. Goiter associated with LS is an uncommon finding. Fever is usually the primary symptom of LS, though trismus and dysphagia may also appear. Even though the majority of the symptoms are localized to the neck, reports of hematogenous dissemination involving distant organs have been made [2].

## Case presentation

A 54-year-old female presented with high-grade fever for 20 days, along with progressive odynophagia for the same duration. The patient also gave a history of gradually increasing painful swelling in the neck for 15 days. On general examination, the patient was afebrile, her pulse rate was 108 beats per minute, and her blood pressure was 100 by 70 millimeters of mercury. On local examination, there was intense congestion at the posterior pharyngeal wall with bilaterally enlarged and congested tonsils.

**Figure 1:**
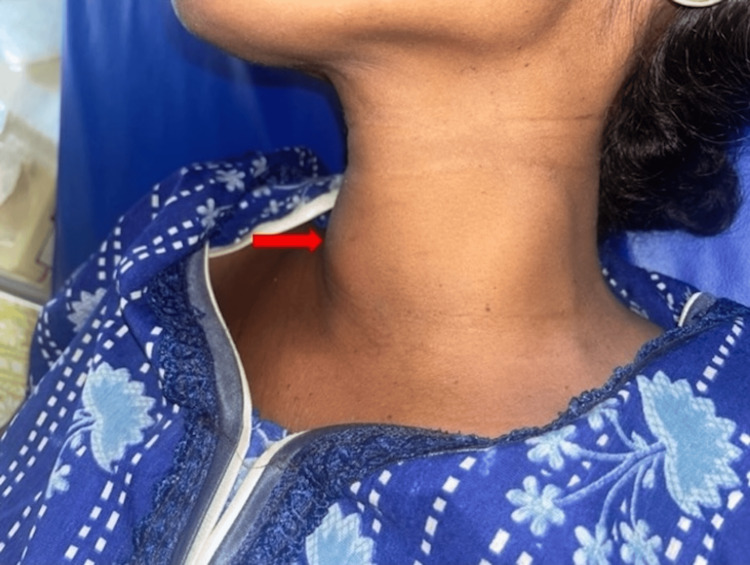
Midline anterior neck swelling on presentation (red arrow)

Examination of the neck revealed a midline swelling, which was approximately 5 cm x 4 cm in size, moved with deglutition and did not move with protrusion of the tongue as shown in Figure 1. It seemed to involve both the lobes of the thyroid. It was warm on touch, tender on superficial palpation, and firm in consistency. The skin above the swelling was normal. There were palpable jugulo-digastric and jugulo-omohyoid lymph nodes which were firm in consistency.

The systemic examination of the cardiovascular system, gastrointestinal system, respiratory system, and nervous system did not reveal any abnormality. On the basis of history and examination, a provisional diagnosis of subacute thyroiditis with a differential diagnosis of thyroid abscess was made. The patient was started on intravenous amoxicillin with clavulanate 1.2 gm twice daily along with other non-steroidal anti-inflammatory drugs like diclofenac and ibuprofen and conservative management. The laboratory workup was performed which revealed raised total leukocyte counts and normal thyroid function as shown in Table 1.

**Table 1:** Laboratory parameters of the patient

Investigation	Normal range	Patient's value
Hemoglobin	12-14 mg/dl	12.3 mg/dl
Total leukocyte count	5,000-11,000 cells/cumm	14,700 cells/cumm
Platelet	1.5-4 lacks/cumm	3.56 lacks/cumm
Hematocrit	36-48%	40.6%
Prothrombin time	11.9	12
International normalized ratio	1	1
Urine routine microscopy	-	Normal
Free triiodothyronine	2.77-5.27 pg/ml	3.99 pg/ml
Free thyroxine	0.78-2.19 ng/dl	2.09 ng/dl
Urea	7-17 mg/dl	30 mg/dl
Creatinine	0.5-1.04 mg/dl	1.1 mg/dl
Sodium/potassium	137-145/3.5-5.1 mmol/l	130/3.7 mmol/l
Alkaline phosphatase	38-126 U/L	125 U/L
Alanine transaminase	16-35 U/L	40 U/L
Aspartate aminotransferase	16-35 U/L	57 U/L
Albumin	3.5-5 g/dl	3.8 g/dl
Total bilirubin	0.2-1.3 mg/dl	0.6 mg/dl
Magnesium	1.6-2.3 mg/dl	2.2 mg/dl
Random blood sugars	70-200 mg/dl	108 mg/dl
Thyroid-stimulating hormone	0.465-4.68 micro IU/ml	0.749 micro IU/ml
Oropharyngeal swab culture/sensitivity	-	Growth of gram-positive bacteria in clusters (*Staphylococcus aureus*)

Ultrasonography of the neck was performed to evaluate the swelling, which reported an ill-defined solid cystic lesion in bilateral lobes of the thyroid (Thyroid Imaging Reporting and Data System-3 (TI-RADS-3)), thrombus present in the IJV of the left side, and reactive lymph node present in the left jugular lower pole.

**Figure 2:**
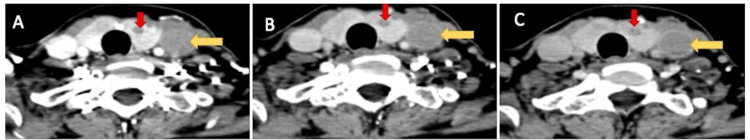
CT scan of the neck arterial phase (A), venous phase (B), and delayed phase (C) showing a filling defect in the left IJV suggesting thrombosis (yellow arrows) and enlarged thyroid gland with non-enhancing hypodense nodule within (red arrows) CT: computed tomography; IJV: internal jugular vein

A computed tomography scan (CT scan) of the neck was done to confirm the diagnosis, and it was suggestive of multiple well-defined non-enhancing hypodense lesions in bilateral lobes of the thyroid with intraluminal filling defect along the course of the left brachiocephalic and IJV, suggestive of thrombosis as shown in Figure 2.

**Figure 3:**
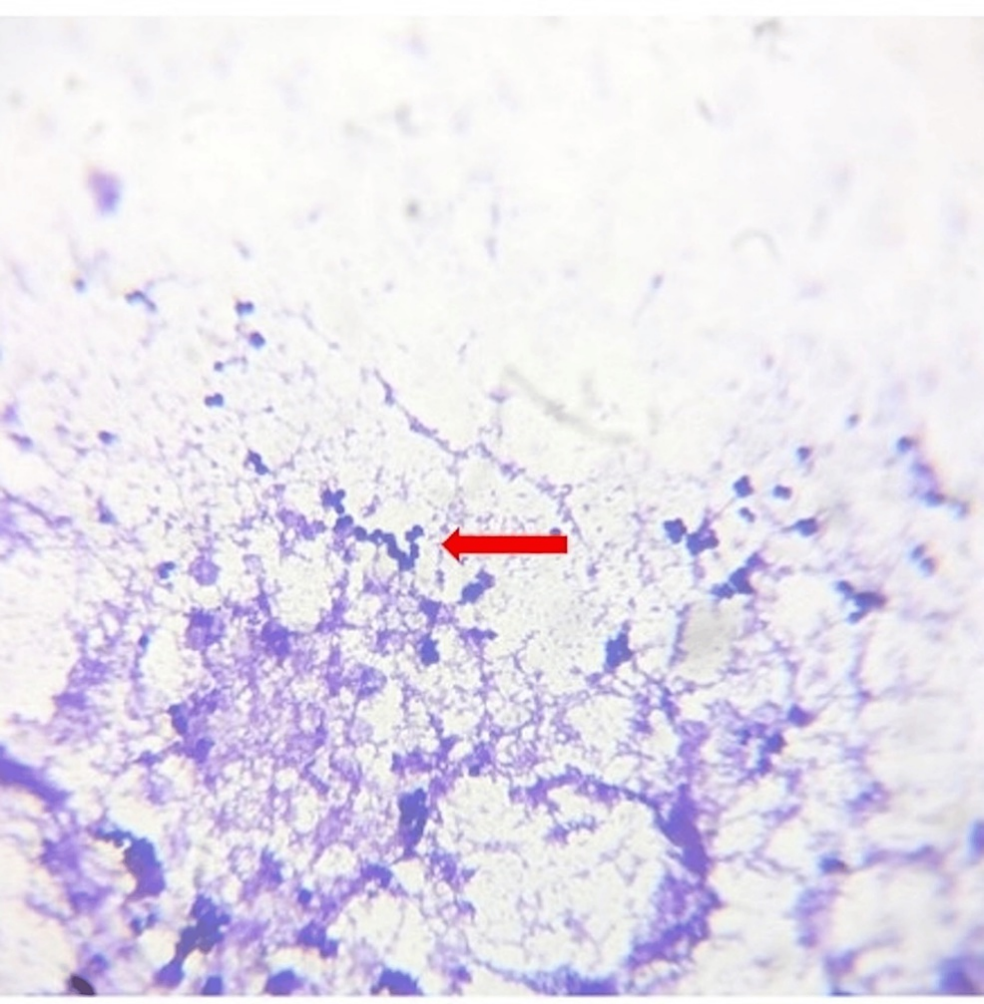
Gram-positive cocci seen on an electron microscope (oil emersion) shown by red arrow

The patient's oropharyngeal swab microscopy and culture sensitivity were done which revealed the growth of gram-positive bacteria seen in clusters like *Staphylococcus aureus* which was sensitive to piperacillin, tazobactam, and linezolid as shown in Figure 3. She underwent fine needle aspiration cytology for the thyroid swelling, and the report was suggestive of nodular colloid goiter (Category II as per the Bethesda system).

After the diagnosis of LS was made, the patient's antibiotic was shifted from amoxicillin to piperacillin and tazobactam with metronidazole for six weeks. The patient was also started on low molecular weight heparin for six weeks, along with the antibiotics. Over two weeks, the patient's pain decreased and the swelling also regressed in size. The patient was discharged on day 14 with oral antibiotics and anticoagulation and was asked to follow-up after four weeks.

## Discussion

LS is an uncommon illness that affects only one instance per million people annually, largely because of the widespread use of antibiotics [2]. Thyroid, pharyngeal, and cervical lymph node infections are the most often mentioned causes. It has been documented that LS can manifest as goiter [3]. In this instance, the patient had a fever with odynophagia and acute anterior neck swelling. A goiter or enlarged thyroid gland was discovered when the swelling was assessed and evaluated. When the patient's thyroid swelling was investigated by fine needle aspiration cytology, the results suggested nodular colloid goiter.

As Lee et al. mentioned in their article Lemierre's Syndrome: A Forgotten and Re-emerging Infection [4], although the prevalence of LS has declined since the introduction of antibiotics, it has been reported more frequently in recent times, particularly in the last 20 years. One reason for this phenomenon is that the practices of prescribing antibiotics have grown more conservative, which has led to an increase in the number of patients who are not receiving adequate treatment for their bacterial infections and are, therefore, more vulnerable to the complications of LS [5].

After LS diagnosis, the initial prognosis remains skeptical because of the high death rate (4-25%), which persists until the present [6]. Depending on the clinical scenario, the duration of treatment can last anywhere from two to six weeks [7]. Early introduction of wide-spectrum antibiotic therapy with anaerobic coverage can improve the prognosis of disease. Extended antibiotic therapy should be used in instances complicated by abscess formation, necrotizing fasciitis, or mediastinitis. Surgical drainage and debridement are also recommended in these situations [8].

The role of anticoagulation is controversial because there are few randomised clinical trials; it's unclear how long anticoagulation medication should be used and whether it should be used at all. This is likely because LS is a rare condition. Nevertheless given the current state of knowledge, the recommended course of treatment is six to 12 weeks of anticoagulation [8] as prescribed for this patient.

The most frequent complication of LS is septic embolism, which might lead to secondary abscesses in the brain or lung, septic pericarditis in the heart, or even tamponade in extreme cases. Organisms commonly involved in this are *Fusobacterium necrophorum*, often known as *Fusobacterium* species, an anaerobe germ of the oropharyngeal sphere which is typically identified by cultures in young patients [9]. *Klebsiella pneumoniae* is more frequently seen in elderly adults with poorly controlled diabetes, and other anaerobic bacteria like *Streptococcus* and *Staphylococcus* are also seen [10].

Early diagnosis is crucial for suspected LS to avoid sepsis and decelerate the disease's progression; yet, due to the condition's benign course and lack of knowledge, diagnosis is frequently delayed. Simple echography, duplex scanning of the cervical area, phlebography, and CT scan all can be used to get a definitive diagnosis [11]. CT with contrast is the most helpful of them for diagnosis; it can reveal filling defects, oedema of the soft tissues, and the mere thrombus itself inside the IJV, as is evident in this case.

## Conclusions

LS is an uncommon yet extremely fatal illness with catastrophic complications. It generally affects the younger population, but regardless of the presence of sepsis, anyone exhibiting signs or symptoms of IJV thrombophlebitis due to oropharyngeal infection should be investigated. Diagnosis should be confirmed with contrast-enhanced CT scans, chest radiographs, and blood cultures. The organism isolated in this case was *Staphylococcus aureus*. Physicians, while managing such oropharyngeal infections not responding to empirical treatment or with immunosuppressed individuals, should investigate patients for LS to prevent the fatal complications that could happen with the same.
